# Knockdown of FBI-1 Inhibits the Warburg Effect and Enhances the Sensitivity of Hepatocellular Carcinoma Cells to Molecular Targeted Agents *via* miR-3692/HIF-1α

**DOI:** 10.3389/fonc.2021.796839

**Published:** 2021-11-12

**Authors:** Juan Liu, Chao Yang, Xiao-Mei Huang, Pan-Pan Lv, Ya-Kun Yang, Jin-Na Zhao, Si-Yuan Zhao, Wan-Jun Sun

**Affiliations:** ^1^ Department of Hematology, PLA Rocket Force Characteristic Medical Center, Beijing, China; ^2^ Department of Neurosurgery, Beijing Huicheng Medical Research Institute, Beijing, China

**Keywords:** the factor that binds to inducer of short transcripts-1, advanced hepatocellular carcinoma, microRNA, HIF-1α, aerobic glycolysis/Warburg effect, molecular targeted agents resistance

## Abstract

The transcription suppressor factor FBI-1 (the factor that binds to inducer of short transcripts-1) is an important regulator of hepatocellular carcinoma (HCC). In this work, the results showed that FBI-1 promoted the Warburg effect and enhances the resistance of hepatocellular carcinoma cells to molecular targeted agents. Knockdown of FBI-1 *via* its small-interfering RNA (siRNA) inhibited the ATP level, lactate productions, glucose uptake or lactate dehydrogenase (LDH) activation of HCC cells. Transfection of siFBI-1 also decreased the expression of the Warburg-effect-related factors: hypoxia-inducible factor-1 alpha (HIF-1α), lactate dehydrogenase A (LDHA), or GLUT1, and the epithelial–mesenchymal transition-related factors, Vimentin or N-cadherin. The positive correlation between the expression of FBI-1 with HIF-1α, LDHA, or GLUT1 was confirmed in HCC tissues. Mechanistically, the miR-30c repressed the expression of HIF-1α by binding to the 3′-untranslated region (3′-UTR) of HIF-1α in a sequence-specific manner, and FBI-1 enhanced the expression of HIF-1α and HIF-1α pathway’s activation by repressing the expression of miR. By modulating the miR-30c/HIF-1α, FBI-1 promoted the Warburg effect or the epithelial–mesenchymal transition of HCC cells and promoted the resistance of HCC cells to molecular targeted agents.

## Introduction

As an important transcription inhibitor (the transcription suppressor), the positive regulator of human malignancies/pro-oncogene (positive regulator/pro-oncogene) FBI-1 (factor that binds to inducer of short transcripts-1) is produced by *Zbtb7A* (zinc finger and BTB domain protein family 7A) and plays important roles in cancerous cells’ proliferation or metastasis (1, 2). The FBI-1 was also named as other names: (1) osteoclast-derived zinc finger (OCZF), (2) leukemia-related factor (LRF) (3–5), and (3) Pokemon (POK erythroid myeloid oncogenic factor) (6, 7). Recently, FBI-1 has been considered as not only the positive regulator of hepatocellular carcinoma (HCC) but also the inducer of resistance of HCC cells to chemotherapies (8). Therefore, it is urgent to extend our knowledge about FBI-1 in HCC cells’ resistance to molecular targeted agents.

The aerobic glycolysis/Warburg effect has been considered as an important feature of cancer cells featured with the elevated glucose uptake and lactate production, especially HCC, and often exhibits the aberrant metabolism feature characterized by the high level of glycolysis and glucose uptake even in the abundant oxygen condition (9–11). *Via* the aerobic glycolysis/Warburg effect, HCC and other kinds of cancerous cells could facilitate the higher proliferation, the alteration of tumor microenvironment, or the resistance of cells to antitumor strategies. Hypoxia-inducible factor-1 alpha (HIF-1α), the most important regulator of hypoxia-related mechanisms, is often overexpressed in human cancers and associated with the poor prognosis (12–16). HIF-1α promotes the progression and malignant characteristics *via* its target genes participating in the cellular survival, tumor angiogenesis, aberrant metabolism, and therapeutic resistance (17–19). Therefore, HIF-1α is a promising target for HCC treatment.

The previous publications showed that FBI-1 could promote the resistance of HCC cells to chemotherapies *via* P53 pathways (8). In the present work, our results, for the first time, revealed that FBI-1 induced the aerobic glycolysis/Warburg effect of HCC cells by enhancing the expression of HIF-1α and its target genes. For the mechanisms, FBI-1 increased the expression of HIF-1α in HCC cells *via* decreasing the expression of miR-3692-5p, which is an miRNA targeting the 3′-untranslated region (the 3’UTR) of HIF-1α. Our research not only expands the understanding of the function and mechanism of FBI-1 but also helps to provide more options for HCC treatment.

## Materials and Methods

### The Clinical-Related Materials, Plasmids, and Cell Culture Lines

A total of advanced HCC patients’ clinical specimens derived complementary DNA (cDNA) samples were gifts from Dr. and Prof. Yingshi Zhang of Shenyang Pharmaceutical University (20). The cell lines used in the present work included the hepatic non-tumor cell line (L-02) and the HCC cell lines MHCC97-H, HepG2, or MHCC97-L. These cells were purchased from a type culture resource of the Chinese government: the Type Culture Collection of the Chinese Academy of Sciences, Shanghai, China. These cells were cultured by using the Dulbecco’s modified Eagle’s medium (DMEM, Invitrogen, Thermo, Waltham, MA, USA) with 10% fetal bovine serum (FBS, Thermo). The expression plasmids or the small-interfering RNA (siRNA) plasmids of FBI-1 were gifts from Prof. and Dr. Mingyang Li of General Hospital of Chinese PLA (21) and purchased from Vigene Corporation, Jinan City, Shandong Province, China. The overexpression of miR-3692-5p was achieved by preparing the full length sequences of has-pre-miR-3692 (sequence of the precursor molecule of human miR-3692) as lentivirus.

### Antitumor Agents/Molecular Targeted Agents

The molecular targeted agents Sorafenib, Regorafenib, Lenvatinib, or Cabozantinib were purchased from the Selleck Corporation, Houston, TX, USA. For the cellular experiments, the organic solvents dimethyl sulfoxide (DMSO) was simply use to dissolve the pure powder of molecular targeted drugs and then diluted with DMEM that did not contain serum or only contained about 0.5% serum. For animal experiments, organic solvents such as DMSO, PEG400, and Tween 80 were used to dissolve the molecularly targeted drug powder and then diluted with sterile PBS (22–25).

### Quantitative Polymerase Chain Reaction and Biochemical Analysis

For the cell based assays, the HCC cells were cultured and transfected with vectors and harvested. For the animal experiments, the subcutaneous tumor tissues were collected and grinded by using the liquid nitrogen. The RNA samples from the cells or the tumor tissues were extracted and reverse transcribed into cDNA according to the manufacturer’s instructions (Thermo Fisher Scientific, Waltham, MA, USA) (26–28). Then, the cDNA samples were analyzed by quantitative PCR (qPCR) according to the manufacturer’s instructions (Thermo Fisher Scientific, Waltham, MA, USA) and methods described in previous publications (26–28). Primers used in the present work were listed as follows: (1) FBI-1, forward sequence, 5’-GCAACATCTGCAAGGTCCGCTT-3’; reverse sequence, 5’-TCTTCAGGTCGTAGTTGTGGGC-3’; (2) miR-3692-5p, the reverse-transcription primer, 5’-GTCGTATCCAGTGCGTGTCGTGGAGTCGGCAATTGCACTGGATACGACCAGTAT-3’; forward sequence, 5’-CCTGCTGGTCAGGAGTGGATACTG-3’; reverse sequence, 5’-CAGTGCGTGTCGTGGAGT-3’; (3) HIF-1α, forward sequence, 5’-TATGAGCCAGAAGAACTTTTAGGC-3’; reverse sequence, 5’-CACCTCTTTTGGCAAGCATCCTG-3’; (4) GLUT1, forward sequence, 5’-TTGCAGGCTTCTCCAACTGGAC-3’; reverse sequence, 5’-CAGAACCAGGAGCACAGTGAAG-3’; (5) LDHA, forward sequence, 5’-GGATCTCCAACATGGCAGCCTT-3’; reverse sequence, 5’-AGACGGCTTTCTCCCTCTTGCT-3’; (6) N-cadherin, forward sequence, 5’-CCTCCAGAGTTTACTGCCATGAC-3’; reverse sequence, 5’-GTAGGATCTCCGCCACTGATTC-3’; (7) E-cadherin, forward sequence, 5’-GCCTCCTGAAAAGAGAGTGGAAG-3’; reverse sequence, 5’-TGGCAGTGTCTCTCCAAATCCG-3’; and (7) Vimentin, forward sequence, 5’-AGGCAAAGCAGGAGTCCACTGA-3’; reverse sequence, 5’-ATCTGGCGTTCCAGGGACTCAT-3’. The biochemical analysis, including the lactate and ATP level, glucose uptake, or LDH activation, was conducted following the methods described in the previous studies (29–31).

### Patients’ Survival Analysis or the Correlation Analysis

For the survival analysis, the endogenous expression of FBI-1 or HIF-1α was examined by qPCR, and the patients were divided into two groups, namely, the FBI-1/HIF-1α high group or the FBI-1/HIF-1α low group, according to the median values (31, 32). Survival analysis is carried out in combination with the clinical information of the patients, and the clinical significance of the expression of FBI-1 or HIF-1α is determined by the survival curves, the overall survival (OS), or the time to progress (TTP) of patients in the high and low expression groups (33, 34).

For the correlation analysis, the endogenous expression level of FBI-1, HIF-1α, or the downstream genes of HIF-1α was determined. A scatter plot was drawn based on the expression of FBI-1 and HIF-1α. Each dot represented a tumor tissue. The expression of FBI-1 was taken as the abscissa, and the expression level of HIF-1α, etc. was taken as the ordinate. The correlation between two genes was determined by linear regression analysis (35, 36).

### Western Blot Experiments

The hepatic cells (including the non-tumor cell line L-02, or the HCC cell lines HepG2, MHCC97-H, MHCC97-L, BEL-7402, SMMC-7721, or Hu7) were cultured and directly harvested for the Western blot experiments. Moreover, the subcutaneous tumor tissues were harvested and grinded with liquid nitrogen for the Western blot (37, 38). Protein samples obtained from the cultured cells or the tumor tissues were analyzed by sodium dodecyl sulfate–polyacrylamide gel electrophoresis (SDS-PAGE) and trans-printed into polyvinylidene difluoride (PVDF) membranes. After SDS-PAGE or trans-print, the PVDF membranes were blocked by bovine serum albumin (BSA) [5% w/v diluted with Tris-buffered saline with Tween 20 (TBST)] at 37°C for 2 h. Then, the membranes were incubated with primary antibody (anti-SREBP-1 FBI-1, anti-HIF-1α, or anti-β-actin antibodies; all antibodies were purchased from Abcam Corporation, UK) and the secondary antibody [the horseradish peroxidase (HRP)-coupling antibodies, Abcam]. The blots were visualized with an ECL kit (Amersham Biosciences, Piscataway, NJ, USA).

### The MTT and Cell Survival Assays

The HCC cells were transfected with vectors and treated with agents (39, 40). After 48 h treatment of the agents, the MTT experiments were performed according to previous publications. The relative number of cells were revealed by the absorbance values [optical density (OD), 490 nm] at a wavelength of 490 nm. Then, the inhibition rates or the *IC_50_* values of the agents on HCC cells were measured according to the OD values (41, 42).

### The Subcutaneous Tumor Model of HCC Cells in Nude Mice

The subcutaneous tumor model of HCC cells was established in the nude mice model (43–45). The usage of animal-related materials and the methods of animal experiments were reviewed and approved by the Animal Ethic Committee of PLA Rocket Force Characteristic Medical Center, Beijing, China. The nude mice were purchased from the Si-Bei-Fu Corporation, Beijing, China. The HCC cells were cultured and transplanted subcutaneously into the subcutaneous position of nude mice. The mice received the molecular targeted agent Sorafenib *via* by the oral administration, and the agents were administered one time every 2 days. After 21 days of treatment (about 10 times Sorafenib treatments), the nude mice were collected, and the subcutaneous tumors were harvested. The tumor volumes, tumor weights, and the inhibitory rates according to tumor volumes or tumor weights were measured.

### Intrahepatic Tumor Models/Liver *In Situ* Models in Nude Mice

The intrahepatic tumor models/liver *in situ* models in nude mice were prepared according to the methods descripted by Wei et al. and Meng et al. (46, 47). The HCC cell lines, MHCC97-H cells or MHCC97-L cells, which were transfected with plasmids were mixed with the medical hydrogel and adhered onto the nude mice’s liver surface by using the surgical operation following the methods described by Li et al. or Meng (47, 48). Three to 4 days after the surgical operation, mice received 3 mg/kg dose of agents *via* oral administration once per 2 days. After 21 days of treatment (10 times treatment), mice were screened by micro-PET experiments to confirm the tumor nodules in the nude mice’s liver organs (49). Next, the mice were harvested, and the liver organs were collected. Then, the liver organs were analyzed by H&E staining. The images of micro-PET or H&E staining were quantitatively analyzed by ImageJ software (50).

### Statistical Analysis

Statistical analysis in the present work was performed by using Bonferroni’s correction without two-way ANOVA (SPSS 9.0, IBM, Armonk, NY, USA). The IC50 values of antitumor agents were calculated by the Origin software (version No.: 6.1; OriginLab, Northampton, MA, USA). A *p* < 0.05 was considered statistically significant between groups.

## Results

### FBI-1 Induced the Warburg Effect in HCC Cells

**Figure 1:**
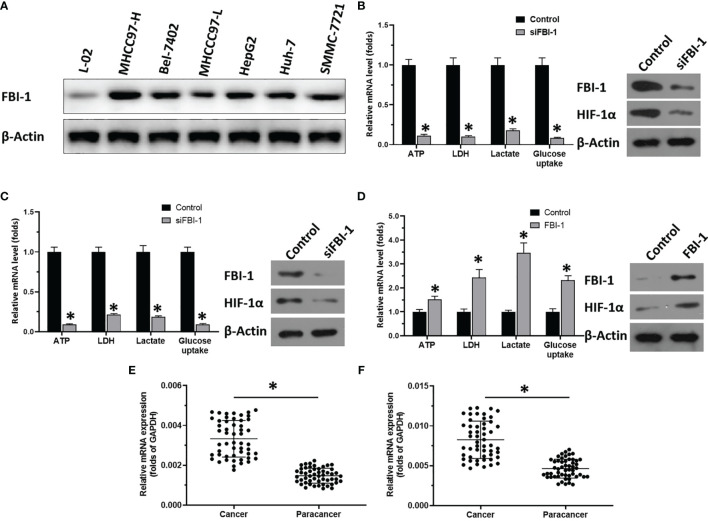
FBI-1 promotes the Warburg effect of HCC cells. **(A)** The endogenous protein level of FBI-1 in hepatic cells. **(B–D)** The effect of FBI-1 on HCC cells’ Warburg effect. **(E, F)** The expression of FBI-1 or HIF-1α. *p < 0.05 versus with control group or siFBI-1 groups **(B–D)**; *p < 0.05 **(E, F)**.

First, to examine the effect of FBI-1 on HCC cells, the endogenous level of FBI-1 in hepatic cell lines was examined by Western blot. As shown in **Figure 1A**, the expression level of FBI-1 in HCC cells was significantly higher than that in L-02 cells. Among the selected HCC cells, the expression level of FBI-1 in MHCC97-H cells is very high, while the expression level of FBI-1 in MHCC97-H cells is very low. Therefore, we will further study to knock down the expression of FBI-1 in MHCC97-H cells and HepG2 and overexpression of FBI-1 in MHCC97-L cells.

As shown in the results in **Figure 1B**, knockdown of FBI-1 *via* its siRNA repressed the Warburg effect of HCC cells: transfection of FBI-1 siRNA decreased the lactate and ATP level, glucose uptake, or LDH activation in MHCC97-H cells. Similar results were obtained from HepG2 cells (**Figure 1C**). Moreover, overexpression of FBI-1 promoted the Warburg effect of HCC: transfection of FBI-1 vectors increased the lactate and ATP production, glucose uptake, or LDH activation in MHCC97-L cells (**Figure 1D**). Therefore, FBI-1 promotes the glycolysis or Warburg effect in HCC cells.

### FBI-1 Enhanced the Expression of HIF-1α

The above results showed that FBI-1 promotes the Warburg effect of HCC cells. The results in **Figures 1B** **–D** indicate that overexpression of FBI-1 enhanced the expression of HIF-1α, whereas knockdown of FBI-1’s expression decreased the expression of HIF-1α. Moreover, as shown as **Figures 1E, F**, the expression of FBI-1 or HIF-1α is much higher in HCC clinical specimens compared with the paired non-tumor tissues.

**Figure 2:**
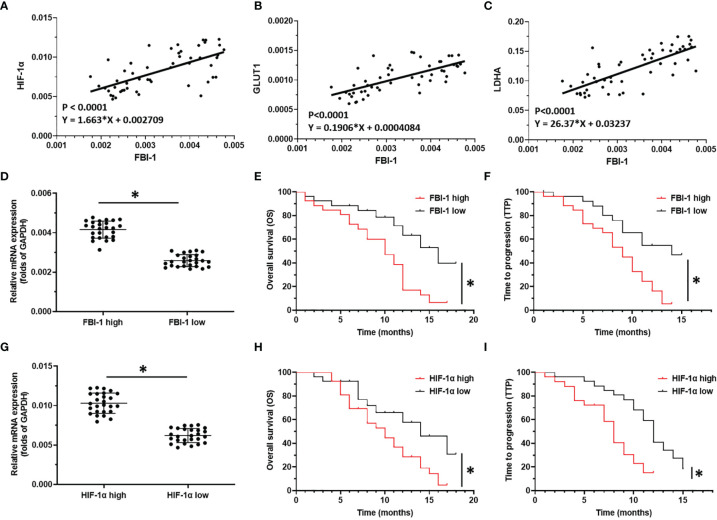
The clinical significance of FBI-1/HIF-1α axis in HCC. **(A–C)** The correlation between FBI-1 with HIF-1α-pathway-related factors. **(D–F)** The correlation between FBI-1 with advanced HCC patients who received sorafenib. **(G–I)** The correlation between HIF-1α with advanced HCC patients who received sorafenib. *p < 0.05.

To further examine the effect of FBI-1 on HIF-1α, the correlation between FBI-1 with HIF-1α in HCC clinical specimens was determined. As shown in **Figures 2A–C**, the expression of FBI-1 was positively related with the expression of HIF-1α (p < 0.0001; Y = 1.663*X + 0.002709) and two typical downstream genes of HIF-1α, GLUT1 (p < 0.0001; Y = 0.1906*X + 0.0004084) or LDHA (p < 0.0001; Y = 26.37*X + 0.03237). Therefore, the possible mechanism for FBI-1 to promote the Warburg effect of HCC cells is to enhance the expression of HIF-1α and its downstream genes.

### FBI-1 and HIF-1α Level Are Negatively Correlated With the Prognosis of HCC Patients Received Molecular Targeted Agents Sorafenib

To further reveal the clinical significance of FBI-1/HIF-1α axis in HCC, the correlation between FBI-1 and HIF-1α with the prognosis of HCC patients who received the molecular targeted agent Sorafenib was shown. As shown in **Figures 2D–I** and **Tables 1**, **2**, the prognosis of advanced HCC patients with low FBI-1 (**Figures 2D** **–F**) or HIF-1α (**Figures 2G** **–I**) levels was much better compared with the patients with high levels of FBI-1 or HIF-1α: the TTP and OS of the high FBI-1 or HIF-1α group were shorter compared to that of the low FBI-1 or HIF-1α group (**Tables 1**, **2**). Therefore, FBI-1 and HIF-1α levels are negatively correlated with the prognosis of HCC patients who received molecular targeted agent Sorafenib.

**Table 1:** FBI-1 expression and clinical outcome of Sorafenib treatment.

	FBI-1 mRNA expression		p
	High (n = 26)	Low (n = 26)	
TTP	9.0	14.0	0.003
	7.1-11.0 (M)	10.1-13.3 (M)	
OS	10.0	15.0	0.001
	7.2–12.8 (M)	12.1–17.6 (M)	

TTP, time to progress; OS, overall survival; M, months.

**Table 2:** HIF-1α expression and clinical outcome of Sorafenib treatment.

	HIF-1α mRNA expression		p
	High (n = 26)	Low (n = 26)	
TTP	8.0	12.0	0.005
	6.9–9.1 (M)	10.9–13.1 (M)	
OS	10.0	14.0	0.032
	6.8–13.2 (M)	8.8–19.1 (M)	

TTP, time to progress; OS, overall survival; M, months.

### FBI-1 Enhanced the Expression of HIF-1α *via* Repressing the Expression of miR-3692-5p in HCC Cells

**Figure 3:**
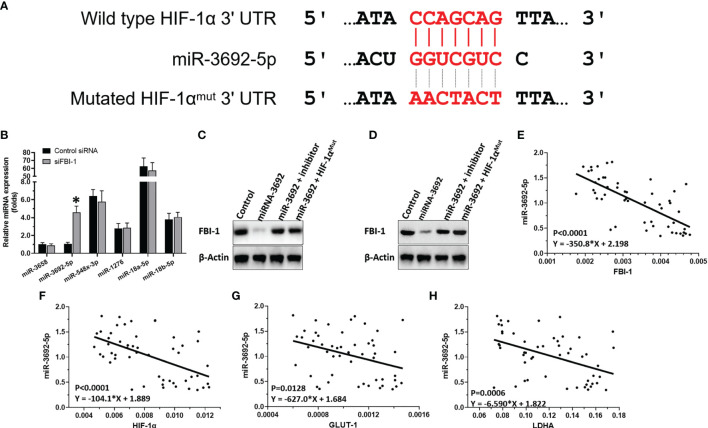
FBI-1 on miR-3692-5p/HIF-1α. **(A)** miR-3692-5p targets on HIF-1α’s 3’-UTR. **(B)** The effect of FBI-1 on the miRNAs on HIF-1α. **(C, D)** The effect of miR-3693-5p on HIF-1α in MHCC97-H or HepG2 cells. **(E–H)** The correlation between miR-3692-5p with FBI-1, HIF-1α, LDHA, or GLUT1. *p < 0.05 versus control siRNA with siFBI-1 **(B)**.

To examine the mechanisms of FBI-1 enhancing the expression of HIF-1α, the effect of FBI-1 on the miRNA potentially targeting the 3’-UTR of HIF-1α predicted by miRDB, a typical online tool, with high scores was examined. As shown in **Figure 3A**, miR-3692-5p targets to the 3’-UTR of HIF-1α. **Figure 3B** shows that knockdown of siFBI-1 enhanced the expression of miR-3692-5p and did not affect other miRNAs. Thus, the miR-3692-5p was chosen for the next steps of experiments.

To confirm the effect of miR-3692-5p on the protein expression of HIF-1α, Western blot was performed. As shown in **Figures 3C, D**, miR-3692-5p inhibited the expression of HIF-1α in MHCC97-H or HepG2 cells. Transfection of miR-3692-5p’s inhibitor or the HIF-1α’s vector with the mutated targeting site of miR-3692-5p blocked the inhibitory effect of miR-3692 on HIF-1α’s protein level. Moreover, the expression level of miR-3692-5p was negatively related to the expression level of HIF-1α (p < 0.0001, Y = −104.1*X + 1.889), GLUT1 (p = 0.0128, Y = −627.0*X + 1.684) or LDHA (p = 0.0006, Y = −6.590*X + 1.822) in the HCC clinical specimens (**Figures 3E** **–H**). Therefore, FBI-1 enhanced the expression level of HIF-1α by repressing the expression of miR-3692-5p.

### The *S*pecificity of FBI-1/HIF-1α in HCC Cells

**Figure 4:**
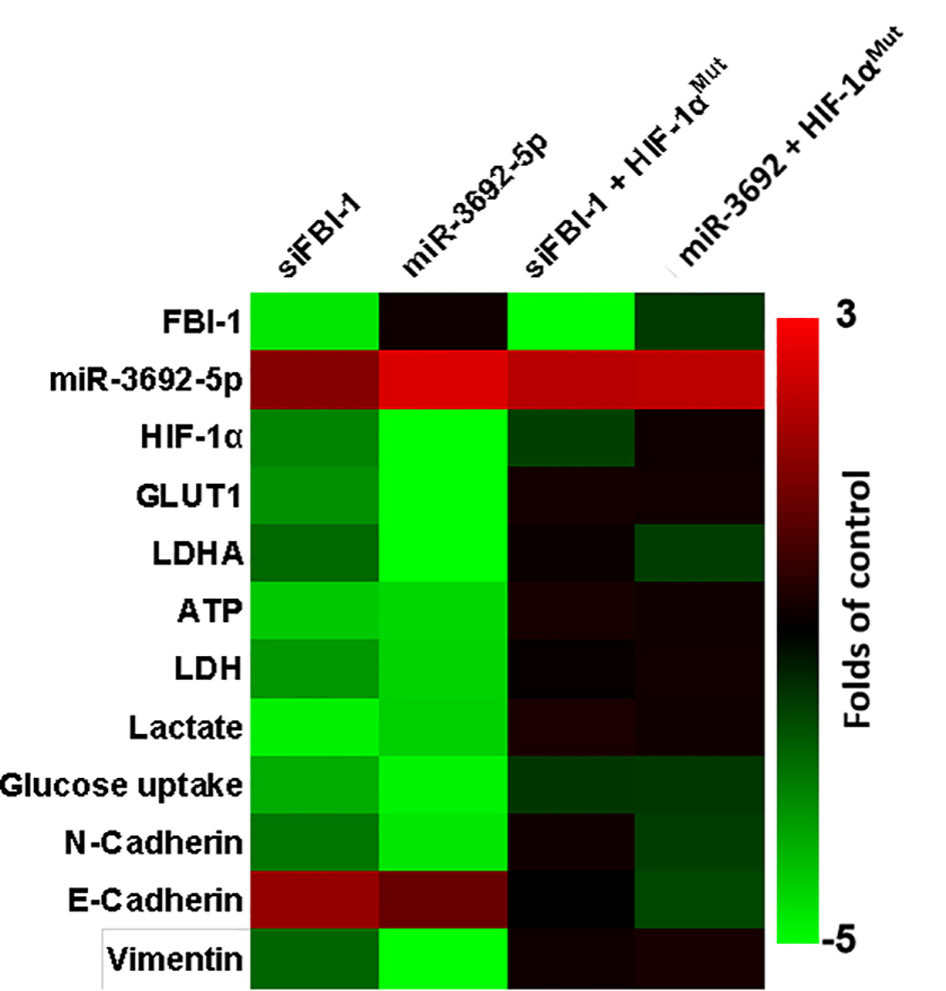
The specificity of FBI-1 miR-3692-5p/HIF-1α. The MHCC97-H cells were transfected with vectors. Cells were measured by qPCR and biochemical analysis. The results are shown as heatmap.

The above results indicated that FBI-1 enhanced the expression level of HIF-1α by repressing the expression of miR-3692. The specificity of FBI-1/HIF-1α in HCC cells was next examined. As shown in **Figure 4**, transfection of siFBI-1 or the miR-3692-5p repressed the expression of HIF-1α-pathway-related factors and inhibited the Warburg effect of MHCC97-H cells. Overexpression of miR-3692-5p did not affect the expression of FBI-1 (**Figure 4**). Transfection of HIF-1α with mutated miR-3692-5p (HIF-1α^Mut^) almost blocked the effect of siFBI-1 or the miR-3692-5p (**Figure 4**); however, HIF-1α^Mut^ did not affect the expression of FBI-1 or HIF-1α. These results further confirmed the specificity of FBI-1/HIF-1α in HCC cells.

### FBI-1 Promotes the Resistance of HCC Cells to Molecular Targeted Agents

To examine whether FBI-1 could promote the resistance of HCC cells to molecular targeted agents, the MTT assays or the nude mice model was used. As shown in **Table 3**, overexpression of FBI-1 enhanced the resistance of MHCC97-L cells to molecular targeted agents: the *IC_50_* values of Sorafenib, Regorafenib, Lenvatinib, or Cabozantinib on MHCC97-L cells were decreased. Moreover, knockdown of FBI-1 *via* its siRNA in MHCC97-H or HepG2 cells enhanced the sensitivity of cells to molecular targeted agents: the *IC_50_* values of Sorafenib, Regorafenib, Lenvatinib, or Cabozantinib on MHCC97-L cells were decreased (**Table 4**).

**Table 3:** FBI-1 induces the resistance of MHCC97-L cells to molecular targeted agents.

Agents	Control	FBI-1	FBI-1 + miR-3692-5p	FBI-1 + miR-3692-5p + HIF-1α^Mut^
	*IC_50_* values (μmol/L)			
Sorafenib	2.22 ± 0.39	10.81 ± 0.60	1.63 ± 0.42	9.36 ± 1.23
Regorafenib	1.83 ± 0.45	12.02 ± 3.70	2.21 ± 0.33	13.34 ± 0.67
Lenvatinib	1.20 ± 0.24	9.01 ± 0.81	1.98 ± 0.41	10.44 ± 0.73
Cabozantinib	1.39 ± 0.27	9.90 ± 1.53	1.40 ± 0.29	9.17 ± 0.39

**Table 4:** siFBI-1 enhances the sensitivity of MHCC97-H cells to molecular targeted agents.

Agents	Control	siFBI-1	siFBI-1 + HIF-1α^Mut^
	*IC_50_* values (μmol/L)		
Sorafenib	1.06 ± 0.22	0.22 ± 0.94	1.54 ± 0.57
Regorafenib	0.97 ± 0.10	0.25 ± 0.08	1.03 ± 0.24
Lenvatinib	0.75 ± 0.32	0.17 ± 0.03	1.01 ± 0.88
Cabozantinib	0.46 ± 0.12	0.10 ± 0.01	0.78 ± 0.14

**Figure 5:**
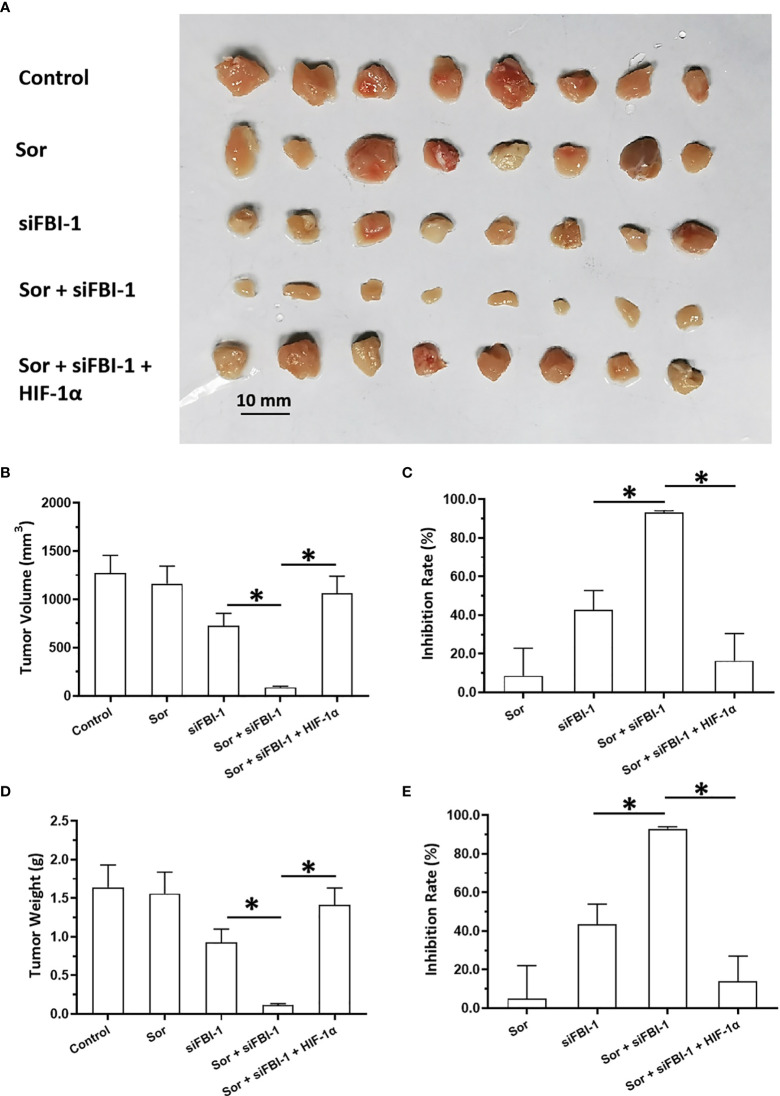
siFBI-1 enhanced the sensitivity of MHCC97-H cells to sorafenib in a nude mice model. The MHCC97-H cells were transfected with vectors and injected into the nude mice to form the subcutaneous tumor models. The mice received the oral administration of 0.5 mg/kg Sorafenib. The results are shown as images **(A)** or the quantitative results **(B–E)**. *p < 0.05.

**Figure 6:**
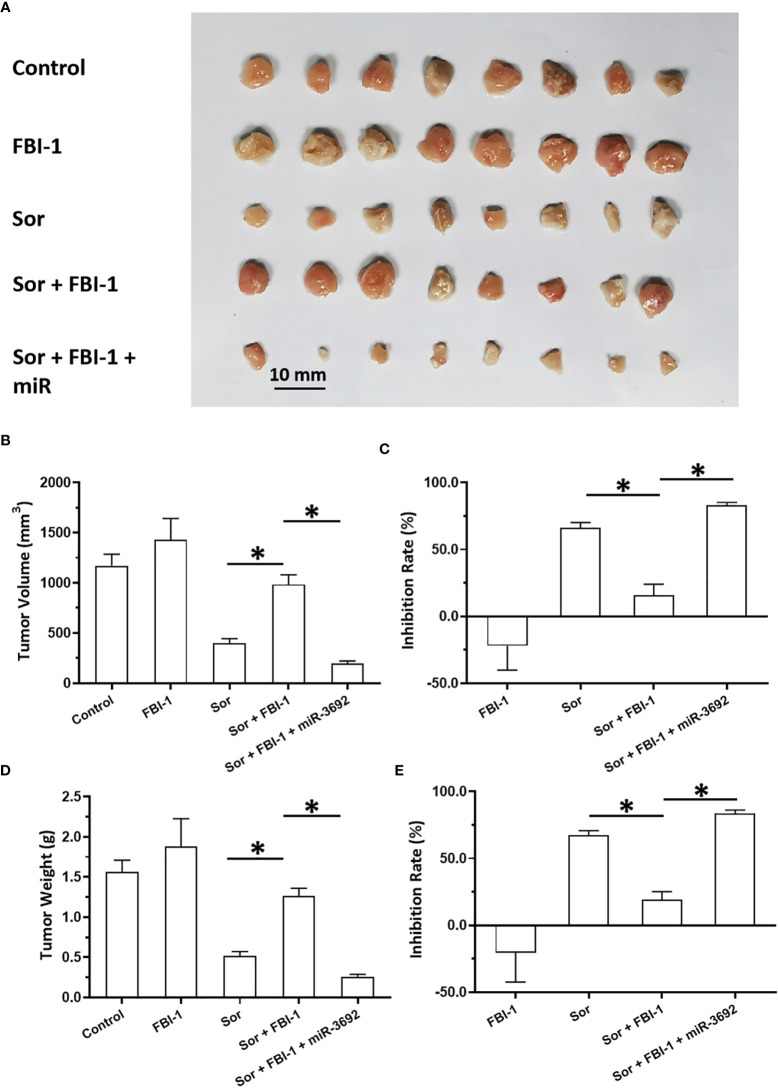
FBI-1 enhanced the resistance of MHCC97-L cells to Sorafenib in a nude mice model. The MHCC97-L cells were transfected with vectors and injected into the nude mice to form the subcutaneous tumor models. The mice received the oral administration of 3 mg/kg Sorafenib. The results are shown as images **(A)** or quantitative results **(B–E)**. *p < 0.05.

The above results were obtained from the cell-based assays. To further examine the effect of FBI-1, the nude mice model was used. As shown in **Figure 5**, 3 mg/kg dose of FBI-1 inhibited the subcutaneous growth of MHCC97-L cells in nude mice, whereas overexpression of FBI-1 enhanced the resistance of MHCC97-L cells to Sorafenib. Overexpression of miR-3692-5p reversed the effect of FBI-1 on Sorafenib. In **Figure 6**, 0.5 mg/kg dose of Sorafenib could not significantly affect the subcutaneous growth of MHCC97-H cells. Knockdown of FBI-1 *via* its siRNA enhanced the antitumor effect of Sorafenib on MHCC97-H cells (**Figure 6**). Overexpression of HIF-1α with mutated miR-3692-5p-targeting sites inhibited the effect of siFBI-1.

**Figure 7:**
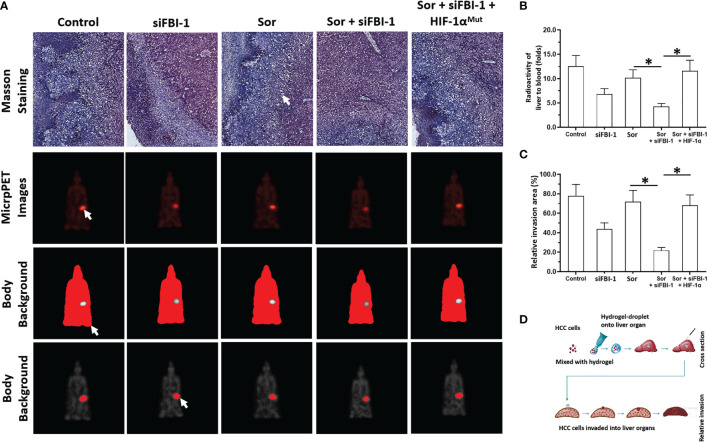
siFBI-1 enhanced the resistance of MHCC97-H cells to Sorafenib in a nude mice model. The MHCC97-H cells were transfected with vectors and injected into the nude mice to form the intrahepatic invasion models. The mice received the oral administration of 0.5 mg/kg Sorafenib. The results are shown as Masson staining **(A)** micro-PET images **(A)**, and the quantitative results of micro-PET images **(A, B)** or Masson staining images **(C)**. The flow chart/model diagram of the intrahepatic invasion model is shown as Panel **(D)**. The white arrow indicated the intrahepatic lesions *via* Masson staining **(A)**, the images of the intrahepatic lesions *via* micro-PET images **(A)**, the body background to quantitatively analyze the micro-PET images **(A)** or the liver region to quantitatively analyze the micro-PET images **(A)**. *p < 0.05.

*Via* the *in vivo*/intrahepatic invasion assays, the MHCC97-H cells could invade into the liver organs of nude mice. Treatment of 0.5 mg/kg dose of Sorafenib did not affect the *in vivo* invasion of MJCC97-H cells into the nude mice’s liver organs (**Figure 7**). Knockdown of FBI-1 *via* its siRNA enhanced the antitumor effect of Sorafenib on the *in vivo* invasion of MJCC97-H cells into the nude mice’s liver organs (**Figure 7**). Therefore, FBI-1 promotes the resistance of HCC cells to molecular targeted agents, and knockdown of FBI-1 *via* its siRNA enhanced the sensitivity of HCC cells to molecular targeted agents.

## Discussion

**Figure 8:**
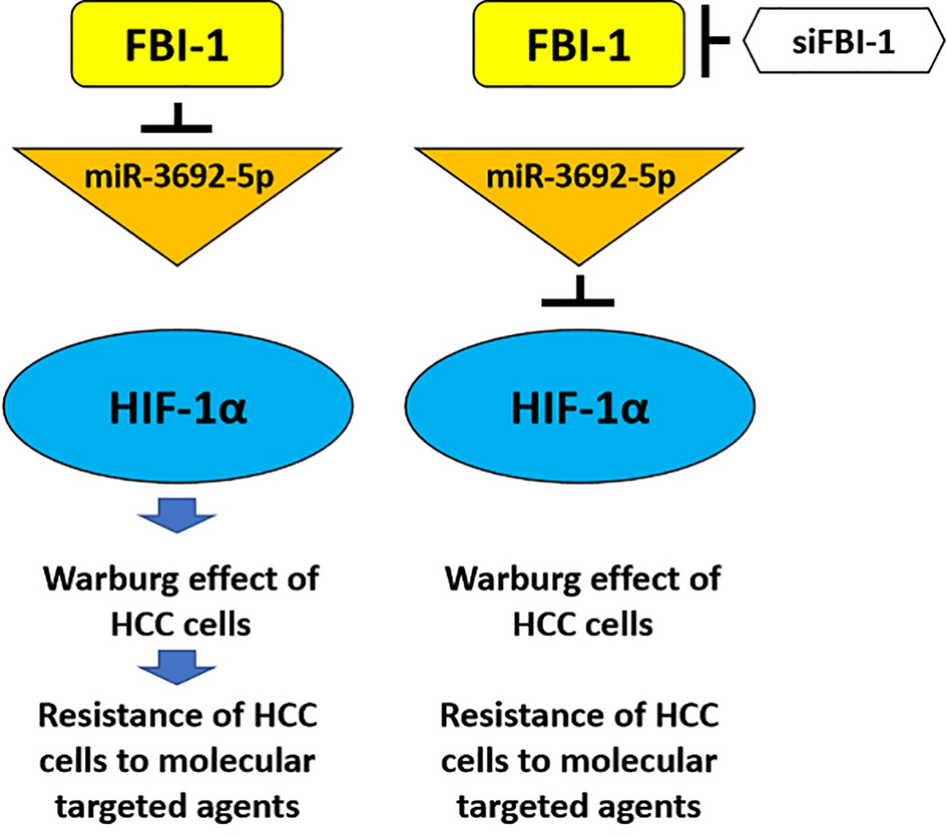
The proposal model of the present work. FBI-1 can downregulate the expression level of miR-3692-5p, thereby causing the upregulation of HIF-1a expression and finally downregulating the sensitivity of HCC cells to molecular targeted drugs by inducing the Warburg effect of HCC cells. The siRNA of FBI-1 is used to inhibit the expression of FBI-1. At this time, the expression level of miR-3692-5p is upregulated, and then by inhibiting the expression of HIF-1α and inhibiting the Warburg effect of HCC cells, it reverses the resistance of HCC cells to molecular targeted drugs.

It is well known that the Warburg effect feature as aerobic glycolysis is not only considered as the common feature of cancer cells, especially HCC, but also alters the microenvironment of tumor lesions related to the proliferation, metastasis, and especially the resistance of cells to antitumor agents. The epithelial–mesenchymal transformation (EMT) process, an important cellular program, which plays important roles in the antitumor resistance, is often related to the alteration of tumor microenvironment induced by the Warburg effect (51, 52). In this study, FBI-1 can upregulate the expression of HIF-1α and its downstream factors, promote the Warburg effect and EMT process of HCC cells, and ultimately cause HCC cells to be resistant to molecular targeted drugs (**Figure 8**). FBI-1 is not only a positive regulator of HCC and other types of malignant tumor cell proliferation but also believed to be able to regulate the resistance of HCC cells to chemotherapeutic drugs. The results of Fang et al. showed that FBI-1 can induce resistance of HCC cells to cytotoxic chemotherapeutics through the P53 pathway (8). For this reason, the results of this study not only expand our understanding of FBI-1 but also provide new enlightenment for HCC treatment.

Furthermore, this study first found that FBI-1 can affect LDH and other biochemical indicators, so as to determine that FBI-1 can affect HCC cell metabolism. Furthermore, FBI-1 can upregulate the expression of HIF-1α, which may be the mechanism by which FBI-1 regulates the metabolism of HCC cells. For the downstream genes of HIF-1α, GLUT1 and LDHA are selected in this study. Increasing evidence has confirmed that the expression of LDHA or GLUT-1 is mediated by the HIF-1α (53–56). Among these two genes, the GLUT1 mediates the uptake of glucose as the key relator of the first step of glycolysis, whereas the LDHA catalyzes the pyruvate into lactate as the last step of glycolysis (57–59). All these indicate that the experimental design of this study is reasonable and provides ideas for similar studies.

At the same time, for the mechanism of FBI-1 in this performance, FBI-1 can play a role by downregulating the expression of miR-3692-5p. FBI-1 is considered to be a transcriptional inhibitor (60, 61). The results of Yang et al. showed that FBI-1 can inhibit the activity of SP1 in triple negative breast cancer (TNBC) cells, downregulate the expression of miR-30c, and then upregulate the expression of PXR, and ultimately accelerate the antitumor drug Olaparib’s metabolism and clearance rate to induce cell resistance (62). Yang et al. (2020) revealed that FBI-1 repressed the miR-30c *via* inhibiting the activation of SP1 and the recruitment of SP-1 to miR-30c’s promoter regions (62). This is also similar to the recognized mechanism of FBI-1: FBI-1 can act as a transcription repressor to downregulate P53 or SP1. To this end, it is necessary to conduct bioinformatics analysis on the sequence of the promoter region of miR-3692-5p and predict which transcription factors can bind to the promoter of miR-3692-5p and the possible interaction between FBI-1 and these transcription factors. Moreover, as the body’s regulatory center for the metabolism and elimination of exogenous drugs and toxicants, PXR expression in hepatocytes and HCC cells is significantly higher than in other types of tissues (63–66). It has been confirmed that PXR also plays an important role in the resistance of HCC cells to molecular targeted drugs (67–70). Therefore, it is of great significance to carry out research on FBI-1 and PXR in HCC cells in the future.

Finally, molecular targeted drug therapy is still an important part of the treatment strategy for advanced HCC (70). Molecular targeted drugs represented by Sorafenib, as multitarget protein kinase inhibitor, cannot only directly inhibit the proliferation of HCC cells but also inhibit the metastasis, invasion, and angiogenesis of HCC cells (71–73). In addition to Sorafenib, some new molecular targeted drugs such as Cabozantinib, Regorafenib, and Lenvatinib were also selected in this study (74–76). The research methods include not only MTT experiments but also a variety of animal models. Therefore, this study not only has scientific research value but also has great clinical value. The HCC cells may be resistant to molecular targeted drugs through a variety of mechanisms: (1) compensation between different signaling pathways (77–79); (2) epithelial–mesenchymal transition (80–82); (3) cancer stem cells (83–85); (4) PXR and other drug metabolism clearance mechanism (63); and (5) Notch pathway and other cell prosurvival and antiapoptotic mechanisms (38, 86–88). This research not only expands our molecular mechanism of HCC cell resistance to molecular targeted drugs but also helps to provide new strategies for HCC treatment.

## Data Availability Statement

The original contributions presented in the study are included in the article/supplementary material. Further inquiries can be directed to the corresponding authors.

## Ethics Statement

The studies involving human participants were reviewed and approved by the ethics committee of PLA Rocket Force Characteristic Medical Center. The patients/participants provided their written informed consent to participate in this study. The animal study was reviewed and approved by the ethics committee PLA Rocket Force Characteristic Medical Center.

## Author Contributions

JL, CY, and W-JS: concept, design, statistics, data collection, manuscript writing, and final approval. X-MH and P-PL: design, statistics, and data collection. Y-KY and J-NZ: concept and data collection. S-YZ: statistics and manuscript writing. JL: statistics and data collection. JL, CY, and W-JS: statistics and data collection. JL and W-JS: concept, design, statistics, data collection, manuscript writing, and final approval. All authors contributed to the article and approved the submitted version.

## Conflict of Interest

The authors declare that the research was conducted in the absence of any commercial or financial relationships that could be construed as a potential conflict of interest.

## Publisher’s Note

All claims expressed in this article are solely those of the authors and do not necessarily represent those of their affiliated organizations, or those of the publisher, the editors and the reviewers. Any product that may be evaluated in this article, or claim that may be made by its manufacturer, is not guaranteed or endorsed by the publisher.
